# The Effects of Psychological Counseling Types on Stress, Athlete Burnout, and Resilience in Athletes: AI Chatbot vs. Face-to-Face Counseling

**DOI:** 10.3390/bs16101805

**Published:** 2026-10-02

**Authors:** Ga-Young Kim, Hye Jin Yang, Chulhwan Choi, Chul-Ho Bum

**Affiliations:** 1 Department of Human Movement Studies and Special Education, Old Dominion University, Norfolk, VA 23529, USA; gkim005@odu.edu; 2 Department of Physical Education, Graduate School, Kyung Hee University, Seocheon-dong 1, Giheung-gu, Yongin-si 17104, Republic of Korea; y0108577@khu.ac.kr; 3 Department of Physical Education, Gachon University, Seongnamdaero, Sujeong-gu, Seongnam-si 13120, Republic of Korea; 4 Department of Golf Industry, College of Physical Education, Kyung Hee University, Seocheon-dong 1, Giheung-gu, Yongin-si 17104, Republic of Korea

**Keywords:** AI chatbot, sports counseling type, sports athletes, stress, athlete burnout, resilience

## Abstract

This study examined the effects of artificial intelligence (AI) chatbot counseling and face-to-face counseling on perceived stress, athlete burnout, and resilience among athletes. In this three-arm quasi-experimental pretest–posttest study, 73 athletes participated in AI chatbot counseling, face-to-face counseling, or a control condition over a 12-week intervention period. Nonparametric longitudinal analyses revealed significant group-by-time interactions. After adjustment for multiplicity, significant group-by-time interactions were observed for perceived stress and reduced sense of accomplishment, a sub-variable of athlete burnout. Perceived stress significantly decreased in the face-to-face counseling group, whereas reduced sense of accomplishment significantly decreased in the AI chatbot counseling group. For supportiveness, a sub-variable of resilience, the group-by-time interaction was significant before multiplicity adjustment but did not remain significant after adjustment. These findings indicate different patterns of change across the counseling conditions and psychological outcomes. Given the quasi-experimental design and baseline differences among the groups, these findings should be interpreted cautiously and further investigation is needed to clarify the potential roles of AI chatbot counseling and face-to-face counseling in supporting athletes’ psychological well-being.

## 1. Introduction

Athletes are exposed to many competitive situations and are required to perform under pressure (Gould et al., 2002). In sports, the result of a competition can be determined by split-second decisions and individual performance, highlighting the importance of athletes’ ability to cope with psychological stress and remain focused on their performance. However, athletes are constantly exposed to various sources of stress, such as competition with peers, pressure to win, and the demands of high-intensity training (Gould & Whitley, 2009; Ntoumanis & Biddle, 1998; Perna et al., 1998). As stress accumulates, the resulting psychological and physical burden can reduce athletes’ self-confidence and interest in sport (Roberts et al., 2019), and ultimately lead to a decline in athletic performance (Arnold et al., 2017; Arnold & Fletcher, 2021). Moreover, athletes who are trying to deal with these stressful conditions but do not achieve their expected goals may experience a reduced sense of accomplishment. People could feel a diminished sense of personal achievement when goals are not met despite pushing their limits or when their efforts are perceived as insufficiently rewarded (Freudenberger, 1977). A persistent sense of reduced accomplishment could contribute to burnout.

Burnout is a state in which an individual loses the motivation to engage in an activity (Harris & Harris, 1984). If a state of burnout is prolonged, it may lead to dropout from sport. The main causes of burnout include sport-related stress and overtraining (Raglin & Morgan, 1989; Smith, 1986; Steinacker & Lehmann, 2002). Another major burden for athletes is the psychological pressure to meet the expectations of others (Schmidt & Stein, 1991). Sport also involves unpredictable situations, so athletes need to adapt and cope effectively with changing environments (Rutter, 2006). In this context, resilience is important for preventing burnout and reducing mental exhaustion among athletes (Galli & Vealey, 2008).

Resilience refers to the capacity to recover and cope with psychologically challenging situations (Reivich & Shatté, 2003). In the context of sport, resilience is important for maintaining sport participation and performance (Egeland et al., 1993). According to Fletcher and Sarkar (2012), high-performing athletes and those who are not exhibit differences in their psychological coping skills. Thus, rather than focusing only on eliminating sources of stress, it is important to understand how athletes perceive and respond to stressful situations.

Negative mental states can affect not only athletes’ performance but also their levels of exhaustion (Cresswell & Eklund, 2006; Weinberg & Gould, 2010). In response, the importance of psychological counseling and psychological skills training (PST) has been emphasized (Orlick & Partington, 1987; Partington & Orlick, 1987; Vealey, 1988). While previous generations of sports coaches and athletes recognized the importance of psychological factors in performance to some extent, they did not use related strategies effectively due to a lack of specialized knowledge and the belief that these characteristics were innate (Kornspan, 2013). Traditional psychological counseling can also be difficult to access continuously because of barriers related to accessibility and cost (Heath et al., 2012; Pereira & Díaz, 2019). Therefore, it is necessary to investigate alternative approaches that can improve accessibility and reduce financial burdens.

In modern society, artificial intelligence (AI) is recognized as an innovative technology across many industries and is being integrated into numerous fields (Oh et al., 2025). One of these technologies is AI chatbots, a conversational artificial intelligence which can interact and converse with humans (Abdul-Kader & Woods, 2015; Laranjo et al., 2018). Conversational AI is an emerging area of mental health treatment with considerable potential, as previous studies have demonstrated that empathy and supportive responses communicated through AI-based conversations can help reduce negative emotions (Ghandeharioun et al., 2016; Paredes et al., 2014). Additionally, chatbots can provide services that can be tailored to individual situations and are accessible at any time and from anywhere (Inkster et al., 2018). Fulmer et al. (2018) suggested that AI chatbots can provide consistent support without imposing a financial burden on users. Recent psychological research has demonstrated that conversational AI chatbots can support human psychological well-being (Korteling et al., 2021). These findings highlight the potential of conversational AI as a tool for providing psychological support.

Many people often struggle to express their feelings openly and honestly. However, research has shown that people feel more comfortable expressing their emotions when interacting with conversational AI because they are aware that their conversational partner is not a real person (Lucas et al., 2014). Artificial intelligence is becoming more common in modern society and the significance of AI chatbots with emotional intelligence is becoming increasingly relevant (Bickmore & Cassell, 2001; Gratch et al., 2007). Research on AI chatbots has primarily focused on psychological counseling in psychiatric treatment (Ghandeharioun et al., 2019) or in the context of sports, on sports medicine and athletic performance analysis (Ramkumar et al., 2022). The importance of psychological skills training and counseling that provide ongoing support for athletes has been emphasized (Strean & Roberts, 1992; Tenenbaum & Bar-Eli, 1992; Vealey, 1994), suggesting a need for more accessible forms of psychological support for athletes. Therefore, it is necessary to investigate whether AI chatbots can serve as an effective alternative or complementary approach to traditional face-to-face psychological support. This study aims to address this need by comparing changes in athletes’ perceived stress, burnout, and resilience across AI chatbot counseling, face-to-face counseling, and control conditions.

## 2. Materials and Methods

### 2.1. Study Participants

The number of participants for this study was calculated using the GPower program (version 3.1.9.7; Heinrich Heine University Düsseldorf, Düsseldorf, Germany). An a priori power analysis was conducted based on a two-way mixed-design repeated-measures ANOVA (within–between interaction). Assuming a medium effect size (Cohen’s *f* = 0.25), an alpha level of 0.05, statistical power of 0.80, three groups, and two measurement occasions (pre- and post-intervention), with a correlation of 0.50 among repeated measures and a nonsphericity correction of ε = 1.00, the analysis indicated a minimum required sample size of 42 participants (14 per group). This study employed a three-arm quasi-experimental pretest–posttest design conducted over a 12-week intervention period. Adult athletes were recruited between November and December 2025 using purposive sampling, a non-probability sampling method. Participants were recruited from collegiate, professional, and semi-professional sport settings in South Korea. Participants were eligible for inclusion if they were aged 18 years or older, were currently active athletes competing at the collegiate, professional, or semi-professional level, and were able to participate in the study intervention. Participants were initially recruited without assignment to a specific study condition. They were subsequently allocated to one of three study conditions while considering sex and sport type to improve comparability across groups. Participants’ willingness and availability to participate in AI chatbot or face-to-face counseling were also considered during the allocation process. Participants who did not participate in either counseling intervention were allocated to the control condition. The individual participant was the unit of assignment. A total of 79 participants were recruited and allocated to the AI chatbot counseling group (n = 26), the face-to-face counseling group (n = 28), or the control group (n = 25). Because of the nature of the interventions, participants and intervention providers could not be blinded to the study condition. Outcome data were collected using self-administered questionnaires, administered either online or in person at a designated study location, depending on participants’ availability. Predefined minimum participation requirements were established for each intervention group. Participants were considered insufficiently adherent if they missed three or more AI chatbot sessions or two or more face-to-face counseling sessions. Based on these criteria, three participants in the AI chatbot counseling group and one participant in the face-to-face counseling group were excluded from the final analysis. In the control group, two participants were excluded due to straight-lining response patterns, indicating unreliable responses. Accordingly, the final analysis followed a per-protocol approach and included only participants who met the predefined intervention participation criteria and provided valid outcome data. No adverse events related to either intervention were reported during the 12-week study period. Ultimately, data from a total of 73 participants were included in the analysis. The final sample consisted of 23 participants in the AI chatbot counseling group, 27 in the face-to-face counseling group, and 23 in the control group. The final sample exceeded the minimum required sample size of 42 participants determined by the a priori power analysis. The demographic characteristics of the participants are presented in Table 1, and their distribution across the three study conditions is presented in Table 2.

### 2.2. Design and Intervention Protocols

The AI Chatbot Counseling group utilized a custom GPT configured specifically for this study using OpenAI’s ChatGPT platform (GPT-5.2) to deliver a psychological counseling intervention tailored to athletes. The custom GPT was developed and tested using GPT-5.2 but was configured to utilize the platform’s standard model allocation rather than a pre-specified model version. Therefore, the underlying model used to generate responses may have varied according to the platform’s standard model allocation. Additionally, cross-session continuity was not formally standardized across participants or sessions. However, the chatbot operated under the same predefined instructions throughout the 12-week intervention period, with no modifications made during the intervention. Participants in the AI chatbot counseling group accessed the custom GPT individually through a link provided by the research team. The AI chatbot intervention was conducted over a 12-week period, from 12 January to 4 April 2026. The system prompt was systematically engineered based on cognitive behavioral therapy (CBT), solution-focused brief therapy (SFBT), and Person-Centered principles to mitigate athletic stress and prevent burnout while enhancing psychological resilience. To minimize cognitive fatigue and evaluation anxiety, the chatbot initiated sessions with casual check-ins and provided empathetic responses rather than directly asking about psychological distress. Furthermore, scenarios frequently encountered by athletes, such as performance anxiety, slumps, and burnout, were embedded into the system. The chatbot guided athletes toward concrete micro-actions to rebuild self-efficacy and address a reduced sense of accomplishment in an empathetic conversational style. The complete system prompt used to configure the AI chatbot is provided in Table 3. Sessions were conducted twice a week, lasting 15 to 20 min per session. This intervention duration was established based on a study by Qi (2024), which involved an 8-week intervention where athletes interacted with the Woebot chatbot for 20 min per session. To monitor intervention adherence, participants were requested to submit a confirmation link after completing each counseling session, thereby measuring actual participation. To protect participants’ privacy and reduce potential discomfort associated with disclosing personal psychological experiences, chatbot conversations were not accessible to or monitored by the researchers. Participants were informed that if they experienced psychological deterioration, encountered concerning or inappropriate chatbot responses, or experienced a situation requiring additional psychological support, they should contact the research team.

Face-to-face counseling sessions were conducted in a designated counseling space based on a structured protocol, with specific content flexibly tailored to each athlete’s individual characteristics. The sessions provided structured emotional support and were tailored to individual psychological needs. The counseling protocol was based on PST and incorporated cognitive-behavioral techniques, with core components including stress management, goal setting, and positive self-talk. Sessions generally involved identifying current sport-related stressors, exploring athletes’ cognitive and emotional responses, and developing appropriate psychological strategies that could be applied to training and competition settings. All sessions were conducted by two sport psychology counselors, both with doctoral degrees. An additional research staff member monitored session attendance and intervention participation throughout the 12-week intervention. The control group received no psychological counseling or other study-related intervention during the 12-week study period. Participants in this group completed the same outcome assessments as the intervention groups at pre-intervention and post-intervention but did not participate in either AI chatbot or face-to-face counseling sessions during the intervention period. The AI chatbot and face-to-face counseling interventions differed in delivery mode, session frequency, session duration, and therapeutic content. Accordingly, the interventions were designed to represent two distinct psychological counseling approaches rather than identical counseling protocols delivered through different modalities.

### 2.3. Instruments

The three primary outcome domains were perceived stress, athlete burnout, and resilience, which were assessed at pre-test and post-test using previously validated measurement instruments. No separate secondary outcomes were designated. Athlete burnout was assessed across three subscales: emotional/physical exhaustion, reduced sense of accomplishment, and sport devaluation. Resilience was assessed across four subscales: hardiness, persistence, optimism, and supportiveness.

To measure the stress levels of athletes, this study adapted and modified the Perceived Stress Scale (PSS) originally developed by Cohen et al. (1983) and validated in Korean by Park and Seo (2010). It consists of a total of 10 items scored on a single scale.

Next, to assess athlete burnout, this study adapted and modified the Athlete Burnout Questionnaire (ABQ), originally developed by Raedeke and Smith (2001) and translated/adapted by Yoo (2012), to fit the purpose of this research. Specifically, it consists of a total of 14 items across three subscales: emotional/physical exhaustion (5 items), reduced sense of accomplishment (4 items), and devaluation of sports participation (5 items). One item within the reduced sense of accomplishment subscale was reverse-coded.

Finally, resilience in this study was assessed and restructured based on the Resilience Quotient Test developed by Shin et al. (2009) while incorporating the modified items adjusted by Lee (2022) to align with the research objectives. Specifically, it consists of a total of 15 items across four subscales: hardiness (4 items), persistence (4 items), optimism (4 items), and supportiveness (3 items). The selected items retained the wording of the corresponding items in the original measure, with no substantive item-level wording modifications. All items were rated on a 5-point Likert scale ranging from 1 (strongly disagree) to 5 (strongly agree).

### 2.4. Data Analysis

The individual participant was the unit of analysis. Analyses were conducted using the final analytic sample (n = 73), after excluding participants who did not meet the predefined intervention attendance requirements or whose responses were identified as unreliable. This study was systematically designed to comparatively analyze athletes’ stress, athlete burnout, and resilience based on the types of psychological counseling received. Data were collected through a pre-test and a post-test. Data analysis involved frequency analysis, exploratory analysis, reliability analysis, and correlation analysis. To test statistical assumptions, Shapiro–Wilk and Levene’s tests were conducted to evaluate normality and homogeneity of variance, respectively, determining the choice between parametric and non-parametric analyses. To complement the significance tests, relative treatment effects (RTEs) with 95% confidence intervals were reported as nonparametric effect estimates for each group-by-time combination. For follow-up analyses, eta-squared based on the Kruskal–Wallis H statistic (η^2^H) and rank-based effect size *r* for Mann–Whitney U and Wilcoxon signed-rank tests were reported. Statistical analyses were conducted using SPSS Version 29.0 and R version 4.3.2 (RStudio, Boston, MA, USA), with the statistical significance level set at α < 0.05.

## 3. Results

### 3.1. Scale Validity and Reliability

Exploratory factor analyses (EFAs) were conducted using principal axis factoring (PAF) with Varimax rotation to examine the underlying factor structure of the measures. PAF was employed to identify the underlying latent factor structure based on the shared variance among observed variables, rather than to reduce the observed variables based on total variance. Varimax rotation was applied when multiple factors were retained to enhance the interpretability of the factor structure. Across all items, factor loadings exceeded the 0.50 threshold, providing preliminary support for the expected factor structure within the current sample. For perceived stress, the KMO value was 0.908 and Bartlett’s test of sphericity was significant (*p* < 0.001). A single factor was identified, with factor loadings ranging from 0.635 to 0.831 and explaining 54.610% of the total variance. For athlete burnout, the KMO value was 0.849 and Bartlett’s test was significant (*p* < 0.001). Three factors were identified: emotional and physical exhaustion (0.800–0.884), sport devaluation (0.732–0.877), and reduced sense of accomplishment (0.639–0.808), collectively explaining 74.268% of the total variance. For resilience, the KMO value was 0.782 and Bartlett’s test was significant (*p* < 0.001). Four factors were identified: supportiveness (0.786–0.876), hardiness (0.648–0.801), persistence (0.512–0.805), and optimism (0.527–0.690), collectively explaining 63.112% of the total variance (Table 4, Table 5 and Table 6).

Internal consistency was assessed using Cronbach’s alpha. The coefficients were 0.922 for perceived stress; 0.947, 0.868, and 0.925 for emotional/physical exhaustion, sport devaluation, and reduced sense of accomplishment, respectively; and 0.857, 0.847, 0.767, and 0.924 for supportiveness, hardiness, persistence, and optimism, respectively. All coefficients exceeded 0.70, indicating acceptable internal consistency (Nunnally & Bernstein, 1994).

### 3.2. Correlation Analysis for Multicollinearity

This study examined statistically significant correlations between all variables by conducting a correlation analysis (−0.289 to 0.560). Furthermore, the strongest positive correlation was observed between emotional/physical exhaustion and reduced sense of accomplishment (*r* = 0.560, *p* < 0.01), whereas the strongest negative correlation was between reduced sense of accomplishment and supportiveness (*r* = −0.512, *p* < 0.01). Perceived stress, emotional/physical exhaustion, reduced sense of accomplishment, and sport devaluation showed weak to moderate negative relationships (−0.289 to −0.519) with hardness, persistence, optimism and supportiveness. Other positive relationships between all variables presented weak to moderate results (0.255 to 0.560). Table 7 provides a summary of the items used to measure each variable and its respective sub-variables.

### 3.3. Nonparametric Two-Way Mixed-Design ANOVA

Prior to the main analysis, the normality and homoscedasticity of the data were examined to evaluate their suitability for a two-way mixed-design repeated measures ANOVA. First, the Shapiro–Wilk test indicated that while the normality assumption was met for most variables, it was violated for some. Second, Levene’s test for equality of variances showed that the assumption of homoscedasticity was not met for certain variables, as their *p*-values were lower than 0.05. Consequently, because these parametric assumptions were violated, a nonparametric repeated measures analysis was ultimately employed. A two-way Wald-type statistic (WTS) was used within the nonparametric longitudinal framework to directly test the main effects of group and time, as well as the group × time interaction. The analysis was conducted using the nparLD package (version 2.3.1) in R. Stress, athlete burnout, and resilience were treated as three distinct outcome domains, with no separate secondary outcomes designated. Perceived stress was assessed as a single outcome, whereas athlete burnout comprised three dimensions (emotional/physical exhaustion, reduced sense of accomplishment, and sport devaluation), and resilience comprised four dimensions (hardiness, persistence, optimism, and supportiveness). To address multiplicity in the Group × Time interaction tests, Holm’s sequential adjustment was applied separately within each multidimensional outcome domain. Specifically, the three athlete burnout dimensions constituted one family and the four resilience dimensions constituted another family. Because perceived stress was assessed as a single outcome, no within-domain multiplicity adjustment was required. Relative treatment effects (RTEs) with 95% confidence intervals were additionally reported as nonparametric effect estimates for each group-by-time combination. The results of the nonparametric two-way mixed-design ANOVA are presented in Table 8, and the corresponding RTEs with 95% confidence intervals are presented in Table 9. Statistically significant interaction effects were found for perceived stress (WTS = 7.93, *df* = 2, *p* = 0.019), reduced sense of accomplishment, a sub-variable of athlete burnout (WTS = 10.75, *df* = 2, *p* = 0.005), and supportiveness, a sub-variable of resilience (WTS = 6.51, *df* = 2, *p* = 0.039). After applying Holm’s adjustment for multiplicity within each outcome domain, the interaction effects remained statistically significant for perceived stress (adjusted *p* = 0.019) and reduced sense of accomplishment (adjusted *p* = 0.014), whereas the interaction effect for supportiveness was no longer statistically significant (adjusted *p* = 0.154). To further examine the significant group-by-time interaction effects, follow-up analyses were conducted using Kruskal–Wallis tests for between-group differences at each time point, followed by pairwise Wilcoxon rank-sum tests with Holm adjustment. Within-group pre–post changes were examined using Wilcoxon signed-rank tests with Holm adjustment.

For perceived stress, significant between-group differences were found at both pre-test, χ^2^(2) = 16.42, *p* < 0.001, η^2^H = 0.206, and post-test, χ^2^(2) = 7.61, *p* = 0.022, η^2^H = 0.080. Pairwise comparisons showed significant differences between the AI chatbot and face-to-face counseling groups at both time points (adjusted *p* < 0.001, *r* = 0.632, and adjusted *p* = 0.009, *r* = 0.414, respectively). Within-group analysis showed a significant reduction only in the face-to-face counseling group, from 3.08 ± 0.47 to 2.75 ± 0.47 (adjusted *p* < 0.001, *r* = 0.795). Correspondingly, the RTE decreased from 0.694 (95% CI [0.626, 0.755]) at pre-test to 0.549 (95% CI [0.469, 0.626]) at post-test in the face-to-face counseling group, whereas relatively little change was observed in the AI chatbot and control groups. For reduced sense of accomplishment, significant between-group differences were observed at both pre-test, χ^2^(2) = 12.47, *p* = 0.002, η^2^H = 0.150, and post-test, χ^2^(2) = 9.18, *p* = 0.010, η^2^H = 0.103. Pairwise comparisons indicated a significant difference between the face-to-face counseling and control groups at pre-test (adjusted *p* = 0.002, *r* = 0.470) and between the AI chatbot and face-to-face counseling groups at post-test (adjusted *p* = 0.005, *r* = 0.439). Within-group analysis showed a significant reduction only in the AI chatbot group, from 2.80 ± 0.90 to 2.24 ± 0.92 (adjusted *p* < 0.006, *r* = 0.647). Correspondingly, the RTE decreased from 0.512 (95% CI [0.421, 0.603]) to 0.348 (95% CI [0.265, 0.441]) in the AI chatbot group.

For supportiveness, a significant between-group difference was found at pre-test, χ^2^(2) = 25.07, *p* < 0.001, η^2^H = 0.330. The Group × Time interaction did not remain statistically significant after Holm adjustment within the resilience outcome domain (adjusted *p* = 0.154). Descriptively, supportiveness increased in the face-to-face counseling group, remained relatively stable in the AI chatbot group, and decreased in the control group. Descriptively, supportiveness increased in the face-to-face counseling group from 3.28 ± 0.99 to 3.78 ± 0.84, remained relatively stable in the AI chatbot group (4.30 ± 0.70 to 4.23 ± 0.69), and decreased in the control group (4.28 ± 0.80 to 3.28 ± 0.99). However, these descriptive patterns should be interpreted cautiously because the interaction did not remain statistically significant after adjustment for multiplicity. The corresponding RTE increased from 0.275 (95% CI [0.206, 0.357]) to 0.391 (95% CI [0.298, 0.492]) in the face-to-face counseling group, remained relatively stable in the AI chatbot group (0.566 [0.482, 0.646] to 0.542 [0.459, 0.623]), and decreased in the control group (0.657 [0.575, 0.731] to 0.569 [0.467, 0.665]). These different patterns of change across groups may explain why the significant between-group differences observed at pre-test were no longer evident at post-test.

Regarding the remaining outcomes, significant main effects of group were found for sport devaluation, persistence, and optimism (all *p* < 0.001), with no significant main effects of time. In contrast, emotional/physical exhaustion and hardiness showed significant main effects of both group (both *p* < 0.001) and time (both *p* = 0.008).

## 4. Discussion

The purpose of this study is to compare and examine stress, athlete burnout, and resilience according to the type of psychological counseling provided to athletes.

First, a statistically significant group-by-time interaction was observed for perceived stress. The face-to-face counseling group showed the largest decrease in mean scores from pre- to post-test, whereas the AI chatbot group showed little change and the control group showed a slight increase. Follow-up within-group analyses indicated a significant reduction only in the face-to-face counseling group. However, between-group analyses revealed that the AI chatbot and face-to-face counseling groups differed significantly at both pre- and post-tests. The decrease observed in the face-to-face counseling group should be interpreted cautiously, particularly given the baseline differences between the groups. This pattern is generally consistent with previous studies demonstrating the potential of psychological interventions to reduce perceived stress (Jones et al., 2020; Vidic et al., 2017). Di Fronso et al. (2022) similarly reported beneficial effects of a face-to-face intervention on stress among athletes. These findings suggest that psychological interventions may have potential for supporting stress management among athletes, who often experience substantial stress in competitive environments (Coelho et al., 2010; Korolev et al., 2021).

Second, a statistically significant group-by-time interaction was found for reduced sense of accomplishment, a sub-variable of athlete burnout. The AI chatbot group showed the largest decrease in mean scores, whereas the control group showed an increase. Within-group analyses demonstrated a significant pre-to-post reduction only in the AI chatbot group. The pattern of between-group differences changed over time: the face-to-face counseling and control groups differed significantly at pre-test, whereas a significant difference between the AI chatbot and face-to-face counseling groups was observed at post-test. These findings are broadly consistent with previous research suggesting that online or non-face-to-face interventions may contribute to improvements in dimensions of burnout (Durden et al., 2023; Wilczyńska et al., 2022). One possible explanation for the observed reduction pattern in the AI chatbot group is the perceived anonymity and lower level of social evaluation that may be associated with AI chatbot interactions. Athletes may experience concerns about stigma or career consequences when disclosing psychological difficulties (Maisarah et al., 2025), whereas AI-based interactions may provide a less socially evaluative environment for expressing personal concerns (Haque & Rubya, 2023; Sowbarnika et al., 2025). Although AI chatbots cannot fully reproduce human empathy and complex interpersonal communication (Seitz, 2024), their accessibility and perceived privacy may facilitate psychological disclosure and support (Peng et al., 2026). These factors were not directly examined in this study and should be considered possible explanations for the findings.

Finally, for supportiveness, a sub-variable of resilience, the group-by-time interaction was statistically significant before adjustment for multiplicity but did not remain significant after Holm adjustment within the resilience outcome domain. Descriptively, supportiveness increased in the face-to-face counseling group, remained relatively stable in the AI chatbot group, and decreased in the control group. After accounting for multiplicity, there was insufficient statistical evidence to conclude that changes in supportiveness differed across the intervention groups. Previous research has suggested that face-to-face interventions and interpersonal support may contribute to resilience (Wang & Liu, 2025). However, the present findings do not provide sufficient statistical evidence to conclude that changes in supportiveness differed across the intervention groups after accounting for multiplicity. Further research with better baseline balance and larger samples is needed to clarify whether counseling approaches are associated with differential changes in supportiveness.

As the role of AI continues to expand, it is increasingly important to examine both the potential benefits and limitations of AI-based psychological interventions. Although the present study showed different patterns of change across psychological outcomes, these findings should be interpreted as exploratory given the nonrandomized allocation procedure and the presence of baseline differences between groups. The observed changes cannot be attributed unequivocally to the counseling interventions or their delivery modalities, as baseline group differences and other unmeasured confounding factors may have contributed to the observed patterns. Further research with improved baseline comparability and larger samples is needed to clarify whether different counseling approaches are associated with differential changes in supportiveness. AI-based interventions may have potential as an additional approach to providing accessible psychological support for athletes. The present findings should be considered preliminary; further research is needed to establish their effectiveness and identify potential risks and limitations.

## 5. Conclusions and Limitations

This study examined changes in stress, athlete burnout, and resilience across AI chatbot counseling, face-to-face counseling, and control conditions. The observed patterns of change across the three groups suggest that different counseling approaches may be associated with changes in different psychological outcomes among athletes. After adjustment for multiplicity, statistically significant group-by-time interactions were observed for perceived stress and reduced sense of accomplishment. Perceived stress significantly decreased within the face-to-face counseling group, whereas reduced sense of accomplishment, a subfactor of athlete burnout, significantly decreased within the AI chatbot counseling group. However, these within-group changes alone provide limited evidence regarding the comparative effectiveness of the interventions. Rather, they should be considered in conjunction with the significant group-by-time interactions and the overall patterns of change across the three groups. For supportiveness, the group-by-time interaction was statistically significant before adjustment for multiplicity but did not remain significant after Holm adjustment within the resilience outcome domain. Accordingly, the observed pattern of change in supportiveness should be interpreted cautiously.

The present findings provide preliminary insights into the potential role of AI chatbot counseling in supporting athletes’ psychological well-being while highlighting the need for further research in sports settings. AI chatbots may provide athletes with immediate and accessible psychological resources while reducing barriers associated with time, location, cost, and discomfort with face-to-face counseling (Bertollo et al., 2021; Britton et al., 2024; Lopes et al., 2024). Rather than replacing human counselors, AI chatbots may therefore serve as complementary tools that extend existing psychological support services (Huțul et al., 2024). Due to the nonrandomized allocation procedure and baseline differences between groups, the observed changes cannot be attributed unequivocally to the counseling interventions or their delivery modalities and should be interpreted as preliminary. Further controlled research is needed to determine whether these potential advantages translate into intervention effects relative to other counseling conditions.

Several limitations should be considered when interpreting the findings. First, the relatively small and heterogeneous sample may have limited statistical precision and the stability of the observed effects. The psychometric properties of the measures were re-examined using this relatively small sample. Although the factor analyses provided preliminary support for the expected factor structures, the limited sample size may have affected the stability and generalizability of the factor solutions. Therefore, these findings should be regarded as a preliminary assessment of the measures within the current sample rather than as definitive psychometric validation. Future studies using larger independent samples are needed to confirm the factor structures. Second, baseline differences were present for several outcomes, potentially reflecting selection bias associated with the nonrandomized allocation process and making it difficult to distinguish intervention-related changes from pre-existing group differences. Third, the AI chatbot and face-to-face counseling interventions differed in session frequency, duration, and therapeutic content. Thus, differences between the intervention groups cannot be attributed solely to the counseling delivery modality and should be interpreted as a comparison of two intervention packages rather than as evidence of the isolated effect of counseling modality. Fourth, chatbot conversations were not monitored by the research team, so inappropriate chatbot responses or adverse psychological experiences may not have been systematically detected during the intervention. Fifth, the domain-specific adjustment did not control the family-wise error rate across all eight outcome tests, so the findings should be interpreted with caution as exploratory. Sixth, the study did not include a long-term follow-up assessment beyond the immediate post-intervention measurement. As a result, it remains unclear whether the observed changes were maintained after the intervention ended. Finally, the use of purposive sampling and the recruitment of athletes exclusively from South Korea may limit the generalizability of the findings to other athlete populations and cultural contexts.

Future studies should employ larger and more diverse samples, stronger randomized designs when feasible, more closely matched intervention conditions, and longer follow-up periods. Such research would help clarify whether AI chatbot-based psychological support produces reliable and sustained effects and determine how it may best complement traditional psychological services for athletes.

## Figures and Tables

**Table 1:** Demographic Characteristics of the Participants.

Category	Item	n	Percentage (%)
Counseling type	AI chatbot	23	31.5 (%)
	Face-to-Face counseling	27	37 (%)
	Control	23	31.5 (%)
Gender	Male	44	60.3 (%)
	Female	29	39.7 (%)
Age	20s	61	83.6 (%)
	30s	12	16.4 (%)
Sport type	Golf	25	34.2 (%)
	Taekwondo	4	5.5 (%)
	Sport shooting	2	2.7 (%)
	Archery	2	2.7 (%)
	Bowling	6	8.2 (%)
	Volleyball	12	16.4 (%)
	Ice hockey	11	15.1 (%)
	Soccer	2	2.7 (%)
	Ski	1	1.4 (%)
	Other	8	11 (%)
Years of experience:	Over 5 years	37	50.7 (%)
	Over 10 years	32	43.8 (%)
	Over 15 years	4	5.5 (%)
Education	High School Graduate or Less	44	60.3 (%)
	Bachelor’s Degree or Less	19	26 (%)
	Master’s/Ph.D. or Higher	10	13.7 (%)
Affiliation	Student-Athletes	35	47.9 (%)
	Professional Athletes	30	41.1 (%)
	Semi-Professional Athletes	8	11 (%)
	Total	73	100 (%)

**Table 2:** Baseline Demographic Characteristics of Participants by Study Condition.

	Characteristics	Total (N = 73)	AI (n = 23)	F2F (n = 27)	Control (n = 23)
Gender, n (%)	Male	44 (60.3)	14 (60.9)	15 (55.6)	15 (65.2)
	Female	29 (39.7)	9 (39.1)	12 (44.4)	8 (34.8)
Sport type, n (%)	Golf	25 (34.2)	9 (39.1)	9 (33.3)	7 (30.4)
	Taekwondo	4 (5.5)	2 (8.7)	2 (7.4)	0 (0.0)
	Sport shooting	2 (2.7)	0 (0.0)	1 (3.7)	1 (4.3)
	Archery	2 (2.7)	1 (4.3)	1 (3.7)	0 (0.0)
	Bowling	6 (8.2)	2 (8.7)	2 (7.4)	2 (8.7)
	Volleyball	12 (16.4)	3 (13.0)	4 (14.8)	5 (21.7)
	Ice hockey	11 (15.1)	4 (17.4)	4 (14.8)	3 (13.0)
	Soccer	2 (2.7)	0 (0.0)	0 (0.0)	2 (8.7)
	Ski	1 (1.4)	0 (0.0)	1 (3.7)	0 (0.0)
	Other	8 (11.0)	2 (8.7)	3 (11.1)	3 (13.0)

**Table 3:** System Prompt Used for the AI Chatbot Counseling Intervention.

Type of Prompt	Content of the Prompt
Role and Goals	You are an artificial intelligence (AI) chatbot providing psychological counseling for athletes. Your primary goals are to alleviate athletes’ stress, prevent burnout, promote a positive psychological state, and strengthen resilience.
Core Counseling Principles	Base your counseling on the principles of cognitive behavioral therapy (CBT), solution-focused brief therapy (SFBT), and person-centered counseling. When an athlete reports anxiety, a performance slump, or burnout, rather than immediately offering solutions, first respond with deep empathy, acknowledge how much effort the athlete has made, and help the athlete comfortably verbalize their emotions. Naturally incorporate sport-related scenarios, such as performance anxiety, slumps, and burnout, into the conversation to provide reassurance that the athlete is not alone in experiencing these difficulties. At the end of the conversation, rather than suggesting ambitious goals, guide the athlete toward a very small and concrete action that can be implemented immediately to help rebuild self-efficacy.
Additional Counseling Principles	1. To minimize cognitive fatigue and evaluation anxiety, do not immediately probe for psychological distress. Always begin the conversation with a casual check-in and an empathic response. 2. Do not probe excessively or make the athlete feel as though they are being interrogated. Do not force the conversation or pressure the athlete to share more than they are comfortable sharing. Maintain a warm and empathic conversational style. 3. Reduce the frequency of direct questions. Rather than asking the athlete a series of questions, provide practically helpful empathic comments and support until the athlete explicitly states that they are struggling or asks for help. 4. Rather than forcing the athlete to disclose information, gently guide them through supportive conversation so that they can naturally reflect on their emotions and identify small, actionable steps.

**Table 4:** Results of exploratory factor analysis on perceived stress.

	1
Perceived stress 9	0.831
Perceived stress 10	0.827
Perceived stress 5	0.766
Perceived stress 8	0.765
Perceived stress 7	0.764
Perceived stress 6	0.741
Perceived stress 2	0.719
Perceived stress 3	0.664
Perceived stress 1	0.649
Perceived stress 4	0.635
Eigenvalue	5.461
Variance (%)	54.610
Cronbach’s alpha	0.922

**Table 5:** Results of exploratory factor analysis on athlete burnout.

	1	2	3
Emotional/Physical Exhaustion 3	0.884	0.228	0.196
Emotional/Physical Exhaustion 1	0.842	0.214	0.235
Emotional/Physical Exhaustion 4	0.841	0.123	0.121
Emotional/Physical Exhaustion 5	0.809	0.049	0.404
Emotional/Physical Exhaustion 2	0.800	0.193	0.308
Sport Devaluation 12	0.085	0.877	0.193
Sport Devaluation 13	0.039	0.870	0.051
Sport Devaluation 11	0.134	0.823	0.041
Sport Devaluation 10	0.287	0.781	0.239
Sport Devaluation 14	0.268	0.732	0.287
Reduced sense of accomplishment 6	0.106	0.266	0.808
Reduced sense of accomplishment 7	0.367	−0.018	0.748
Reduced sense of accomplishment 8	0.343	0.130	0.720
Reduced sense of accomplishment 9	0.238	0.359	0.639
Eigenvalue	3.993	3.718	2.686
Variance (%)	28.522	26.560	19.186
Cronbach’s alpha	0.947	0.868	0.925

**Table 6:** Results of exploratory factor analysis on resilience.

	1	2	3	4
Supportiveness 13	0.876	0.164	0.207	0.195
Supportiveness 15	0.803	0.216	0.294	0.160
Supportiveness 14	0.786	0.191	0.196	0.231
Hardiness 2	0.224	0.801	0.198	0.147
Hardiness 4	0.131	0.721	0.231	0.194
Hardiness 3	0.047	0.678	0.147	0.253
Hardiness 1	0.372	0.648	−0.003	0.250
Persistence 6	0.088	0.144	0.805	0.074
Persistence 7	0.346	0.067	0.765	0.158
Persistence 8	0.141	0.223	0.738	0.141
Persistence 5	0.439	0.153	0.512	0.096
Optimism 10	0.016	0.289	0.150	0.690
Optimism 11	0.118	0.226	0.158	0.669
Optimism 12	0.192	0.057	0.038	0.597
Optimism 9	0.310	0.231	0.082	0.527
Eigenvalue	2.727	2.442	2.378	1.921
Variance (%)	18.177	16.280	15.852	12.804
Cronbach’s alpha	0.857	0.847	0.767	0.924

**Table 7:** Results of correlation analysis.

	1	2	3	4	5	6	7	8
1	1							
2	0.467 **	1						
3	0.512 **	0.560 **	1					
4	0.255 *	0.379 **	0.413 **	1				
5	−0.289 *	−0.361 **	−0.449 **	−0.348 **	1			
6	−0.368 **	−0.519 **	−0.499 **	−0.451 **	0.404 **	1		
7	−0.352 **	−0.370 **	−0.334 **	−0.456 **	0.497 **	0.342 **	1	
8	−0.334 **	−0.464 **	−0.512 **	−0.482 **	0.463 **	0.541 **	0.441 **	1

Note. * *p* < 0.05, ** *p* < 0.01. 1: Perceived stress, 2: Emotional/physical exhaustion, 3: Reduced sense of accomplishment, 4: Sport devaluation, 5: Hardness, 6: Persistence, 7: Optimism, 8: Supportiveness.

**Table 8:** Results of Nonparametric two-way mixed-design ANOVA.

		Group	n	Pre-Test Mean ± SD	Post-Test Mean ± SD	Group Effect	Time Effect	Interaction Effect	Interaction Effect (Adjusted *p*)
Perceived stress		AI chatbot	23	2.32 ± 0.552	2.27 ± 0.508	<0.001 ***	0.148	0.019	0.019 *
		Face-to-Face	27	3.08 ± 0.468	2.75 ± 0.471				
		Control	23	2.59 ± 0.936	2.74 ± 1.07				
Athlete burnout	Emotional/ physical exhaustion	AI chatbot	23	2.71 ± 0.813	2.41 ± 0.817	<0.001 ***	0.008 **	0.925	0.925
		Face-to-Face	27	3.56 ± 0.988	3.27 ± 1.03				
		Control	23	2.92 ± 1.31	2.60 ± 1.10				
	Reduced sense of accomplishment	AI chatbot	23	2.80 ± 0.895	2.24 ± 0.922	0.001 **	0.078	0.005	0.014 *
		Face-to-Face	27	3.36 ± 0.761	3.15 ± 1.00				
		Control	23	2.36 ± 1.06	2.85 ± 1.39				
	Sport devaluation	AI chatbot	23	1.86 ± 0.628	1.84 ± 0.535	<0.001 ***	0.482	0.101	0.201
		Face-to-Face	27	3.22 ± 1.18	3.04 ± 1.28				
		Control	23	1.97 ± 1.08	2.27 ± 0.988				
Resilience	Hardiness	AI chatbot	23	3.42 ± 0.864	3.85 ± 0.547	<0.001 ***	0.008 **	0.224	0.224
		Face-to-Face	27	2.82 ± 0.825	3.25 ± 1.15				
		Control	23	3.75 ± 0.584	3.74 ± 1.11				
	Persistence	AI chatbot	23	3.99 ± 0.605	4.25 ± 0.452	<0.001 ***	0.278	0.085	0.191
		Face-to-Face	27	3.24 ± 0.916	3.32 ± 0.703				
		Control	23	4.30 ± 0.564	4.26 ± 0.737				
	Optimism	AI chatbot	23	3.92 ± 0.576	4.01 ± 0.576	<0.001 ***	0.140	0.064	0.191
		Face-to-Face	27	3.42 ± 0.896	3.32 ± 0.627				
		Control	23	4.15 ± 0.542	3.89 ± 0.794				
	Supportiveness	AI chatbot	23	4.30 ± 0.703	4.23 ± 0.692	<0.001 ***	0.967	0.039	0.154
		Face-to-Face	27	3.28 ± 0.994	3.78 ± 0.842				
		Control	23	4.28 ± 0.795	3.28 ± 0.994				

Note. * *p* < 0.05, ** *p* < 0.01, *** *p* < 0.001.

**Table 9:** Relative Treatment Effects and 95% Confidence Intervals for Study Outcomes by Group and Time.

		Group	n	Pre-Test RTE (95% CI)	Post-Test RTE (95% CI)
Perceived stress		AI chatbot	23	0.360 [0.277, 0.453]	0.345 [0.266, 0.434]
		Face-to-Face	27	0.694 [0.626, 0.755]	0.549 [0.469, 0.626]
		Control	23	0.514 [0.402, 0.624]	0.538 [0.414, 0.657]
Athlete burnout	Emotional/physical exhaustion	AI chatbot	23	0.445 [0.357, 0.536]	0.359 [0.282, 0.443]
		Face-to-Face	27	0.677 [0.591, 0.752]	0.591 [0.502, 0.675]
		Control	23	0.493 [0.378, 0.610]	0.435 [0.341, 0.534]
	Reduced sense of accomplishment	AI chatbot	23	0.512 [0.421, 0.603]	0.348 [0.265, 0.441]
		Face-to-Face	27	0.674 [0.593, 0.747]	0.591 [0.500, 0.677]
		Control	23	0.393 [0.299, 0.495]	0.481 [0.374, 0.590]
	Sport devaluation	AI chatbot	23	0.382 [0.303, 0.468]	0.385 [0.310, 0.465]
		Face-to-Face	27	0.702 [0.617, 0.776]	0.659 [0.569, 0.738]
		Control	23	0.387 [0.290, 0.494]	0.485 [0.394, 0.578]
Resilience	Hardiness	AI chatbot	23	0.482 [0.391, 0.574]	0.624 [0.548, 0.694]
		Face-to-Face	27	0.273 [0.208, 0.350]	0.441 [0.340, 0.547]
		Control	23	0.591 [0.499, 0.676]	0.589 [0.468, 0.701]
	Persistence	AI chatbot	23	0.515 [0.429, 0.600]	0.629 [0.557, 0.696]
		Face-to-Face	27	0.289 [0.212, 0.381]	0.290 [0.229, 0.359]
		Control	23	0.647 [0.558, 0.727]	0.631 [0.524, 0.726]
	Optimism	AI chatbot	23	0.551 [0.466, 0.633]	0.595 [0.511, 0.675]
		Face-to-Face	27	0.370 [0.279, 0.471]	0.305 [0.233, 0.388]
		Control	23	0.645 [0.563, 0.719]	0.534 [0.422, 0.644]
	Supportiveness	AI chatbot	23	0.566 [0.482, 0.646]	0.542 [0.459, 0.623]
		Face-to-Face	27	0.275 [0.206, 0.357]	0.391 [0.298, 0.492]
		Control	23	0.657 [0.575, 0.731]	0.569 [0.467, 0.665]

Note. RTE = relative treatment effect; CI = confidence interval.

## Data Availability

The data presented in this study are available on request from the corresponding authors.
